# Seeing the Light: The Use of Zebrafish for Optogenetic Studies of the Heart

**DOI:** 10.3389/fphys.2021.748570

**Published:** 2021-12-23

**Authors:** Jonathan S. Baillie, Matthew R. Stoyek, T. Alexander Quinn

**Affiliations:** ^1^Department of Physiology and Biophysics, Dalhousie University, Halifax, NS, Canada; ^2^School of Biomedical Engineering, Dalhousie University, Halifax, NS, Canada

**Keywords:** cardiac electrophysiology, opsins, membrane potential, intracellular calcium, genetically encoded voltage indicators (GEVIs), genetically encoded calcium indicators (GECIs)

## Abstract

Optogenetics, involving the optical measurement and manipulation of cellular activity with genetically encoded light-sensitive proteins (“reporters” and “actuators”), is a powerful experimental technique for probing (patho-)physiological function. Originally developed as a tool for neuroscience, it has now been utilized in cardiac research for over a decade, providing novel insight into the electrophysiology of the healthy and diseased heart. Among the pioneering cardiac applications of optogenetic actuators were studies in zebrafish, which first demonstrated their use for precise spatiotemporal control of cardiac activity. Zebrafish were also adopted early as an experimental model for the use of optogenetic reporters, including genetically encoded voltage- and calcium-sensitive indicators. Beyond optogenetic studies, zebrafish are becoming an increasingly important tool for cardiac research, as they combine many of the advantages of integrative and reduced experimental models. The zebrafish has striking genetic and functional cardiac similarities to that of mammals, its genome is fully sequenced and can be modified using standard techniques, it has been used to recapitulate a variety of cardiac diseases, and it allows for high-throughput investigations. For optogenetic studies, zebrafish provide additional advantages, as the whole zebrafish heart can be visualized and interrogated *in vivo* in the transparent, externally developing embryo, and the relatively small adult heart allows for *in situ* cell-specific observation and control not possible in mammals. With the advent of increasingly sophisticated fluorescence imaging approaches and methods for spatially-resolved light stimulation in the heart, the zebrafish represents an experimental model with unrealized potential for cardiac optogenetic studies. In this review we summarize the use of zebrafish for optogenetic investigations in the heart, highlighting their specific advantages and limitations, and their potential for future cardiac research.

## Optogenetics in Cardiac Research

Optogenetics involves the measurement and manipulation of cellular activity using genetically encoded light-sensitive proteins (Deisseroth et al., 2006; Miesenböck, 2009). Originally developed as a set of tools for neuroscience to activate or silence neuronal circuits and observe neuronal activity (Li et al., 2005; Nagel et al., 2005), optogenetic “reporters” (for measurement of membrane potential (Siegel and Isacoff, 1997; Sakai et al., 2001; Ataka and Pieribone, 2002) or intracellular calcium [Ca^2+^] (Miyawaki et al., 1997, 1999; Baird et al., 1999)) and ‘actuators' [for modulation of membrane potential (Nagel et al., 2002, 2003; Boyden et al., 2005)] have now been utilized in cardiac research for over a decade (Entcheva and Kay, 2021). Cardiac optogenetics has had a wide-range of applications, including: (i) all-optical studies of cardiac electrophysiology and high-throughput drug screening; (ii) cell-specific measurement or control to investigate cardiac sub-populations (e.g., myocytes, Purkinje cells, fibroblasts, neurons, and immune cells); (iii) manipulation of cardiac ion channels, G protein- coupled receptor signaling, and energetics; (iv) control of action potential morphology or excitation waves; and (v) cardiac pacing, cardioversion/defibrillation, or arrhythmia termination/ablation. Some of the pioneering studies that applied optogenetics to the heart were performed in zebrafish (*Danio rerio*). Here we provide an overview of the use of zebrafish for cardiac optogenetic studies, highlighting their advantages, limitations, and future potential [for a more general consideration of cardiac optogenetics, please see the recent review by Entcheva and Kay (Entcheva and Kay, 2021)].

## Use of Zebrafish for Optogenetic Studies of the Heart

The zebrafish has become an important integrative animal model for cardiac research, based on its particular advantages as an experimental tool (Table 1) (Gut et al., 2017; Stoyek and Quinn, 2018). The zebrafish offers a fully sequenced genome, which can be easily altered using standard genetic techniques at relatively low cost (in terms of time, effort, and money) (Rafferty and Quinn, 2018; Stoyek et al., in press), and almost every cardiac gene has a human ortholog with analogous function (Howe et al., 2013). This high degree of genetic similarity has permitted researchers to recapitulate a variety of human cardiac diseases in the zebrafish (Bowley et al., in press), which can be studied in a high throughput manner (Kithcart and MacRae, 2018). Functionally, the zebrafish heart has comparable heart rate, action potential morphologies, ion channels (Ravens, 2018), and Ca^2+^-handling proteins (van Opbergen et al., 2018b) to human. Furthermore, it has been shown that cardiac regulatory pathways and mechanisms of both intracardiac (MacDonald et al., 2017) and extracardiac (Stoyek et al., 2016) origin are similar to human, and like the cardiac electrophysiology of the zebrafish (Nemtsas et al., 2010), are often more so than rodents.

**Table 1:** Advantages and limitations of the zebrafish for cardiac optogenetic studies.

**Advantages**	**Limitations**
-Relatively low cost (time, effort, money) -Fully sequenced genome -Relatively easy genetic manipulation -Large number of available transgenic lines -Majority of cardiac genes have human ortholog -Externally developing, transparent embryo -Amenable to high throughput studies -Comparable heart rate, action potential morphologies, ion channels, and calcium-handling proteins to human -Intra- and extracardiac regulatory pathways and mechanisms similar to human -Human cardiac diseases can be recapitulated	- Genome duplication (24% of genes have more than one ortholog) -Small, two-chambered heart -Relatively low-pressure system -Lack transverse tubules -Limited release of calcium from sarcoplasmic reticulum following excitation -Low sensitivity of ryanodine receptors to calcium -Dependence of calcium transient on sarcolemmal influx

Of course, as with any experimental model, there are also limitations to the zebrafish's use (Table 1). The zebrafish heart is small, has only two chambers (one atrium and one ventricle, rather than the four chambers found in human), and generates relatively low pressures (Hu et al., 2001). While over 70% of human genes have at least one zebrafish ortholog, 24% of genes have more than one ortholog (due to a duplication of the zebrafish genome), which can confer redundancy in gene function and confound results of genetic manipulations (Howe et al., 2013). Functionally, while cardiac electrophysiology appears strikingly similar to humans (Vornanen and Hassinen, 2016; Ravens, 2018), there are important differences in cellular calcium cycling (Genge et al., 2016; van Opbergen et al., 2018b). Zebrafish cardiomyocytes have a lack of transverse tubules (Brette et al., 2008), and even though sarcoplasmic reticulum Ca^2+^ levels are much higher in the zebrafish, release of Ca^2+^ from the sarcoplasmic reticulum following excitation (Ca^2+^-induced Ca^2+^ release) appears to be limited (due in part to a low sensitivity of ryanodine receptors to Ca^2+^) (Bovo et al., 2013). As a result, sarcolemmal Ca^2+^ influx is responsible for ~80% of the Ca^2+^ transient in zebrafish cardiomyocytes (compared to 25% in human) (Bovo et al., 2013), although this remains somewhat controversial, as others have shown a strong dependence of contractile force on sarcoplasmic reticulum Ca^2+^ release (Haustein et al., 2015) and the existence of Ca^2+^ sparks with characteristics similar to mammals (Llach et al., 2011). Zebrafish also have a higher sodium-Ca^2+^ exchanger current than in mammals, such that its reverse-mode has been shown to trigger sarcoplasmic reticulum Ca^2+^ release (Zhang et al., 2011).

Considering its use specifically for cardiac optogenetic studies, the zebrafish has a further advantage over other animal models, in that the entire zebrafish heart can be optically accessed *in vivo* in the transparent, externally developing embryo (van Opbergen et al., 2018a) or *in situ* in the relatively small, isolated adult heart (Stoyek et al., 2018), in a manner not possible in mammals. While other non-mammalian models may have a similar advantage (e.g., *Drosophila melanogaster* [Wolf et al., 2006) and *Xenopus laevis* (Warkman and Krieg, 2007)], they are limited in other ways. For instance, while *Drosophila* have been highly utilized for studies of cardiac genetics (Wolf et al., 2006), it is an invertebrate, and differences in the morphology of its heart—which is a tube—limits its applicability for functional studies (Rotstein and Paululat, 2016). The heart of *Xenopus*, on the other hand, is in some ways more anatomically similar to humans than zebrafish—for instance, it has a pulmonary circulation—but there is a limited genetic tool box for their transgenesis (Ishibashi et al., 2008).

Ultimately, the similarities of zebrafish to human, and its particular experimental advantages, have resulted in it being a popular experimental model for optogenetic investigations, both for neuroscience [the brain and nervous system can also be optically accessed in the whole animal (Del Bene and Wyart, 2012; Simmich et al., 2012; Portugues et al., 2013)] and for cardiovascular research (Table 2), which is the focus of this review.

**Table 2:** Previous applications of cardiac optogenetics using zebrafish.

	**Publication**	**Age of study**	**Optogenetic line**	**Application/finding**
**Optogenetic Reporters**	Arnaout et al., 2007	2 dpf	*Tg(cmlc2:gCaMP)^*s*878^*	Investigated mutant model of inherited long QT syndrome, in which loss of rapid delayed-rectifier potassium current (*I*_Kr_) due to *kcnh2* mutation results in mechanical ventricular asystole. Showed lack of calcium (Ca^2+^) waves in the ventricle, suggesting impaired Ca^2+^ cycling.
	Chi et al., 2008	1–21 dpf	*Tg(cmlc2:gCaMP)^*s*878^*	Investigated development of the vertebrate cardiac conduction system and performed a forward genetic screen. Identified four stages of conduction development, which depended on epigenetic mechanical factors, and identified 17 conduction-specific mutations that may represent novel genetic regulators of the cardiac conduction system.
	Tsutsui et al., 2010	2–3 dpf	*Tg(cmlc2:Mermaid)*	Investigated the effect of the histamine H1 receptor blocker astemizole on cardiac excitation. Showed that astemizole caused retrograde propagation from the atrioventricular boundary to the atrium.
	Kirchmaier et al., 2012	5–6 dpf	*Tg(cmlc2:gCaMP)^*s*878^*	Investigated effect of Popeye domain containing gene 2 knock-down. Caused sinoatrial node conduction failure, irregular atrial and ventricular activity, and varying degrees of atrioventricular block.
	Hou et al., 2014	1.5–4 dpf	*Tg(cmlc2:Arch(D95N)- GCaMP5G)* [“*CaViar*”]	Investigated the effects of L-type Ca^2+^ (*I*_Ca, L_) or fast sodium current block. Showed that <4 dpf, cardiac excitation is initiated by Ca^2+^, but by 4 dpf ventricular excitation it is initiated by sodium, while atrial excitation remains Ca^2+^ dependent.
	van Opbergen et al., 2018a	3, 14 dpf	*Tg(myl7:chimeric VSFP-butterfly CY)* *Tg(myl7:Gal4FF;* *UAS:GCaMP6f)*	Investigated effects of pharmacological modulation of the sympathetic nervous system or ion channels on cardiac electrophysiology and Ca^2+^ cycling. Showed that: (i) sympathetic stimulation or block increased or decreased diastolic Ca^2+^ and Ca^2+^ transient amplitudes; (ii) *I*_Kr_ block increased action potential duration; (iii) *I*_Ca, L_ block prevented Ca^2+^ transients, increased ventricular action potential duration, and disrupted atrioventricular conduction; and (iv) differences exist in atrial and ventricular Ca^2+^ cycling during development.
	Salgado-Almario et al., 2020	3 dpf	*Tg(cmlc2:Twitch-1)* *Tg(cmlc2:Twitch-2B)* *Tg(cmlc2:Twitch-4)* *Tg(cmlc2:TN-XXL)*	Tested various novel genetically encoded ratiometric calcium indicators to determine which are the most promising for use in the heart.
**Optogenetic Actuators**	Arrenberg et al., 2010	1–5 dpf	*Tg(E1b:Gal4-VP16^*s*1101*t*^; UAS:NpHR-mCherry^*s*1989*t*^)* *Tg(E1b:Gal4^*s*1101*t*^; UAS:ChR2(H134R)-eYFP^*s*1990*t*^)*	Mapped cardiac pacemaker development. Showed that: (i) at 1 dpf, the pacemaker is at the venous pole; (ii) at 2 dpf, it is more confined to the sinoatrial ring; and (iii) by 3 dpf it is more defined and confined to the dorsal right quadrant of the sinoatrial ring. Further, in 4 dpf embryos, heart rate could be control by pulsed light stimulation of the sinoatrial ring.
	Kopton et al., 2018	3 mpf	*Tg(cmlc2:GtACR1-eGFP)*	Tested whether the heart could be silenced with anion-specific light-activated ion channel. Showed that stimulation applied during the resting (diastolic) phase of the action potential causes depolarization and excitation, but causes repolarization and shortening of the action potential if applied during the (systolic) plateau.

*dpf, days post-fertilization. For further details on the optogenetic zebrafish lines, including the meaning of the abbreviations, see the relevant sections of the text*.

## Studies Utilizing Optogenetic Reporters in the Zebrafish Heart

**Figure 1:**
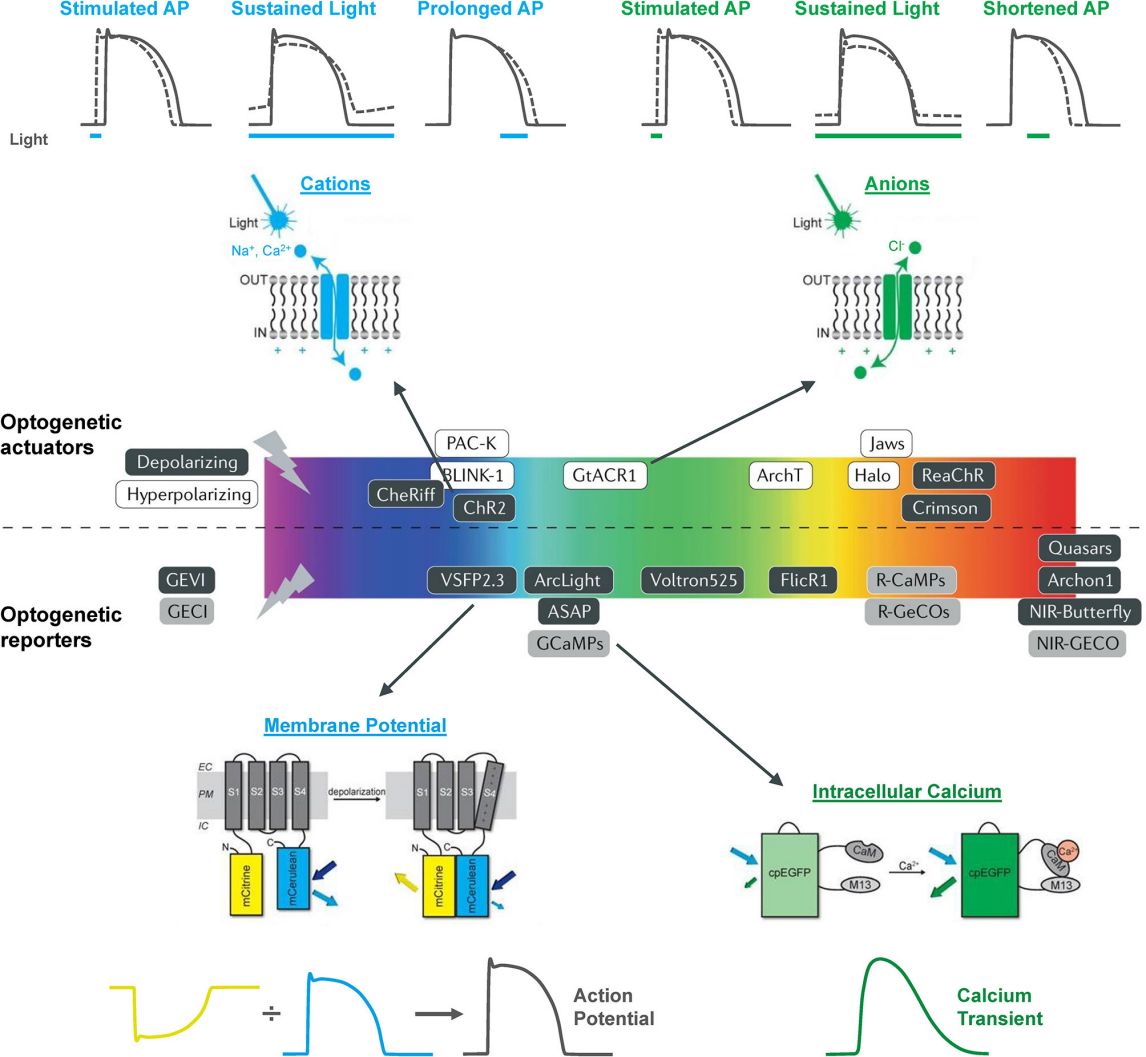
The optogenetic toolbox for measurement and manipulation of cardiac activity. There is a large array of optogenetic actuators and reporters with a broad range of activation spectra available for use in the heart. These comprise light-activated depolarizing (excitatory) and hyperpolarizing (inhibitory) opsins that pass cations, anions, and protons and genetically encoded voltage (GEVI) and calcium (GECI) indicators that can be used to measure membrane potential and intracellular calcium. Opsin schematic from Ferenczi et al. (2019), middle panel from Entcheva and Kay (2021), and GEVI and GECI schematics from van Opbergen et al. (2018a).

The electrical activity of the heart has been optically monitored for decades, well before the emergence of modern optogenetics and the use of functional fluorescent proteins. In the 1970s, Salama and Morad published the first reports of the use of voltage sensitive fluorescent dyes to record cardiac action potentials (Salama and Morad, 1976; Morad and Salama, 1979). Since that time, optical mapping of membrane potential and intracellular Ca^2+^ in the whole heart or isolated tissue and cells has become a “standard” technique in many research labs (Herron et al., 2012; Jaimes et al., 2016; Berenfeld and Efimov, 2019), including studies using zebrafish (Sabeh et al., 2012; Lin et al., 2020). The use of optogenetic reporters (genetically encoded voltage and Ca^2+^ indicators, GEVIs and GECIs, respectively) have additional advantages as they allow for organ-, organelle-, and cell-specific measurements and for *in vitro* and *in vivo* longitudinal studies. There is now a wide array of GEVIs and GECIs available for use in the heart, with a range of excitation and emission spectra, light sensitivity and signal intensity, temporal dynamics, and other properties that dictate their specific use (Figure 1) (Kaestner et al., 2014, 2015; Koopman et al., 2017).

### Genetically Encoded Ca^2+^ Indicators (GECIs)

**Figure 2:**
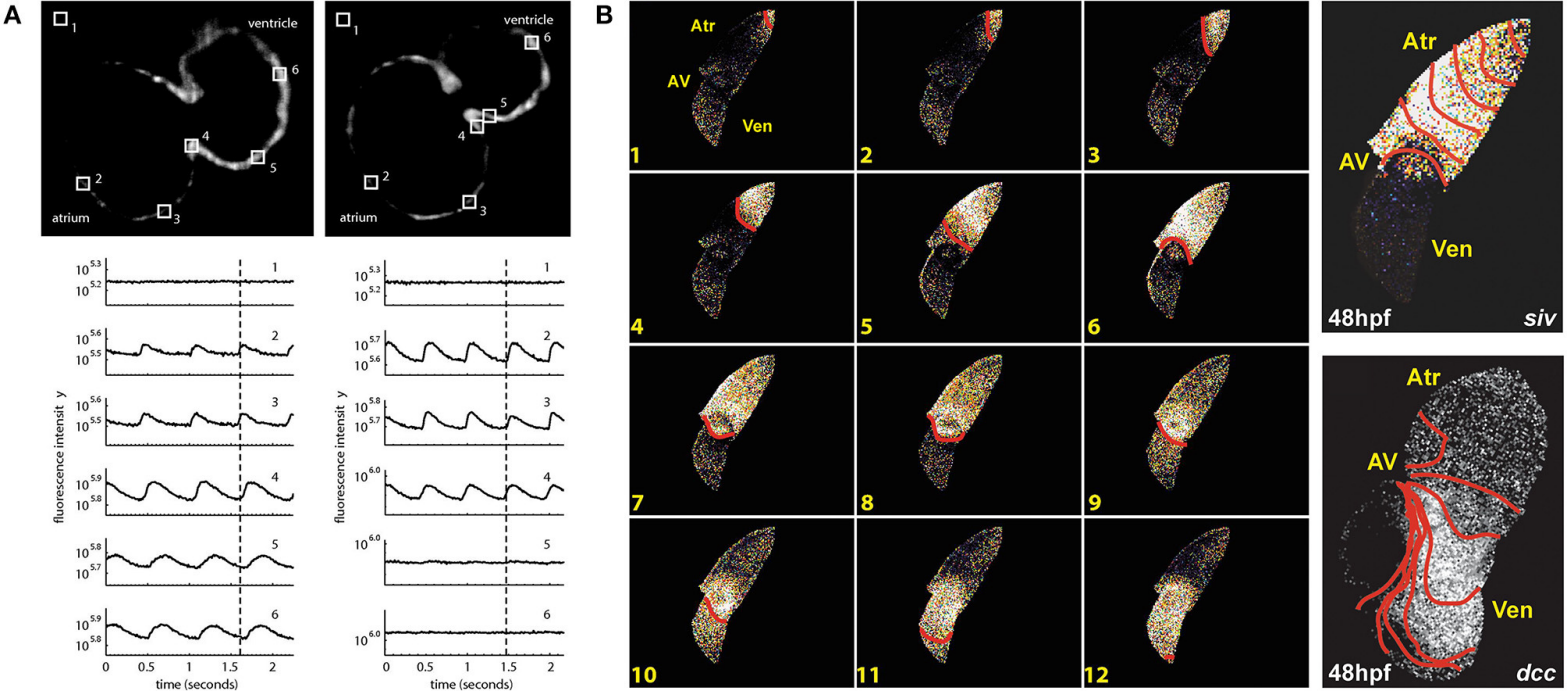
*In vivo* imaging of intracellular calcium using genetically encoded calcium indicators (GECIs) in intact zebrafish embryos. **(A)** Hearts in 48 h post-fertilization (hpf) wild-type embryos with cardiac-specific expression of gGCaMP exhibit atrial and ventricular calcium release (left), while hearts in *kcnh2*^*s*290^ mutants exhibit atrial but no ventricular release (right). Each selected region in the images has a corresponding fluorescence signal plotted below. The dotted lines mark an arbitrary point in time to facilitate comparison across the different signals. From Arnaout et al. (2007). **(B)** In hearts from 48 hpf wild-type embryos, calcium activation travels from the sinus venosus across the atrium (Atr) and ventricle (Ven), with a delay at the atrioventricular (AV) junction (left), while ventricular conduction is absent in *silent ventricle* (*siv)* mutants and disorganized in *dococ*^*s*215, 226^ (*dcc*) mutants (right). Isochronal lines represent 60 ms. From Chi et al. (2008).

The first use of an optogenetic reporter in the heart was described in the early 2000s. This involved GCaMP2, which includes a circularly permutated EGFP within an M13/Calmodulin fusion protein that fluoresces when it binds Ca^2+^, to record Ca^2+^ waves in the isolated mouse heart and in open chest animals (Tallini et al., 2006). This was followed by a study that used GCaMP2 to demonstrate successful engraftment and electrical coupling of embryonic cardiomyocytes with surrounding myocardium in the infarcted mouse heart (Roell et al., 2007). Around the same time, the potential for using zebrafish to image intracellular Ca^2+^ in the *intact animal* was also being realized. The first published report involved the use of a transgenic zebrafish line with cardiac-specific expression of gGCaMP driven by the cardiac myosin light chain 2 (*cmlc2*) gene promoter (*Tg(cmlc2:gCaMP)*^*s*878^). It was used to investigate a transgenic zebrafish model of inherited long QT syndrome in which a loss of rapid delayed-rectifier potassium current (*I*_Kr_) due to a mutation in the *s290* allele of the *kcnh2* gene (*kcnh2*^*s*290^) results in mechanical ventricular asystole (Arnaout et al., 2007). Using selective plane illumination microscopy (SPIM) and excitation-contraction uncoupling with a silent heart cardiac troponin (*tnnt2*) morpholino (to eliminate optical mapping motion artifact associated with contraction), Ca^2+^ transients were measured *in vivo* at various regions of the zebrafish atrium and ventricle in 2 days post-fertilization (dpf) embryos. In wild-type *tnnt2* morpholino-injected zebrafish, repetitive fluorescent waves representing an increase in cytosolic Ca^2+^ during systole were visualized, spreading from the atrium through the atrioventricular junction and into the ventricle (Figure 2A). In contrast, in the *kcnh2*^*s*290^ homozygous mutants, Ca^2+^ waves were visible in the atrium but not in the ventricle (Figure 2A), implying impaired ventricular Ca^2+^ cycling.

The above investigation was followed by a study using the same zebrafish line to map Ca^2+^ waves across the whole heart in intact embryos at various ages to investigate the development of the vertebrate cardiac conduction system (Figure 2B) (Chi et al., 2008). Four distinct stages of conduction development were identified, which corresponded to specific cellular and anatomical changes in the developing heart and were dependent on epigenetic mechanical factors such as hemodynamic flow and contraction. An *in vivo* optical mapping technique was then used as a phenotypic assay to perform a forward genetic screen, which identified 17 conduction-specific mutations (Figure 2B), thought to represent novel genetic regulators of the cardiac conduction system. A similar approach has been applied to other genes whose mutation is known to affect cardiac conduction, such as the Popeye domain containing (*Popdc*) gene family, with morpholino knock down of *popdc2* in 5–6 dpf embryos causing sinoatrial node conduction failure, irregular atrial and ventricular activity, and varying degrees of atrioventricular block (Kirchmaier et al., 2012).

**Figure 3:**
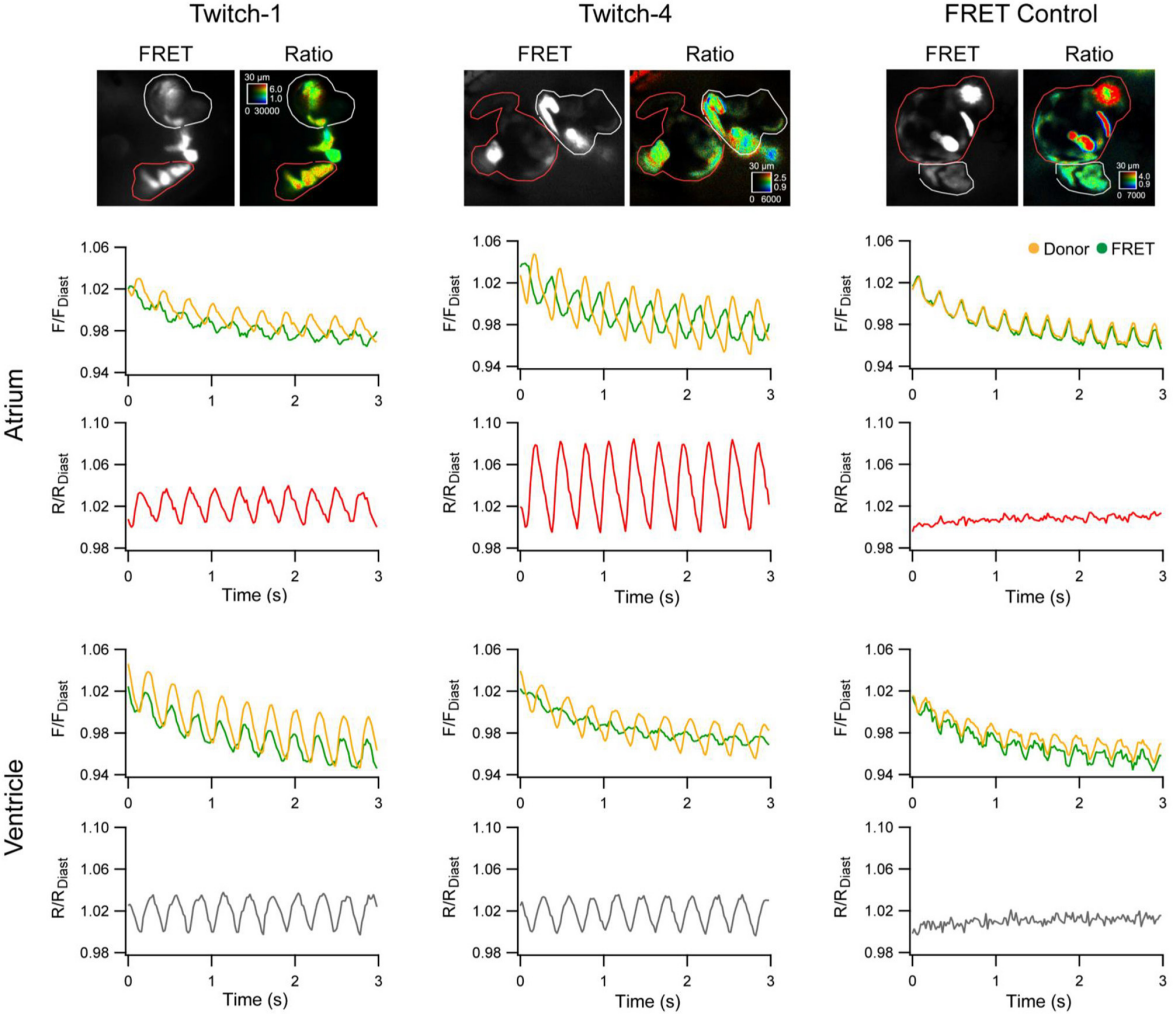
*In vivo* ratiometric intracellular calcium measurements with a genetically encoded calcium indicator (GECI) in intact zebrafish embryos. Ratiometric intracellular calcium signals were acquired with Twitch-1, Twitch-4, or a Förster resonance energy transfer (FRET) construct insensitive to calcium (ECFP-16aa-EYFP) from the atrium (red region-of-interest) and ventricle (white region-of-interest) of 3 days post-fertilization embryos. The change in fluorescence in the donor and FRET channels normalized to the first diastolic period (F/F_Diast_; upper graphs in atrium and ventricle) and their ratio (R/R_Diast_; lower graphs in atrium and ventricle) are shown. From Salgado-Almario et al. (2020).

More recently, zebrafish have been used to help in the assessment of novel GECIs for cardiac-specific applications, such as those with a ratiometric readout, which is useful for assessing absolute changes in Ca^2+^ and to help correct for the motion artifact that occurs with optical mapping in the beating heart. Four available ratiometric Förster resonance energy transfer (FRET)-based GECIs with varying Ca^2+^-binding affinity (TN-XXL, Twitch-1, Twitch-2B, and Twitch-4) were transiently expressed in the hearts of zebrafish embryos (driven by the *cmlc2* promoter) and kinetic parameters of atrial and ventricular Ca^2+^ transients were measured at 3 dpf under various conditions. Ultimately, this revealed that Twitch-1 and Twitch-4 are the most promising for use in the heart, based on their greater sensitivity, faster kinetics, and higher affinity for Ca^2+^ (Figure 3) (Salgado-Almario et al., 2020).

### Genetically Encoded Voltage Indicators (GEVIs)

**Figure 4:**
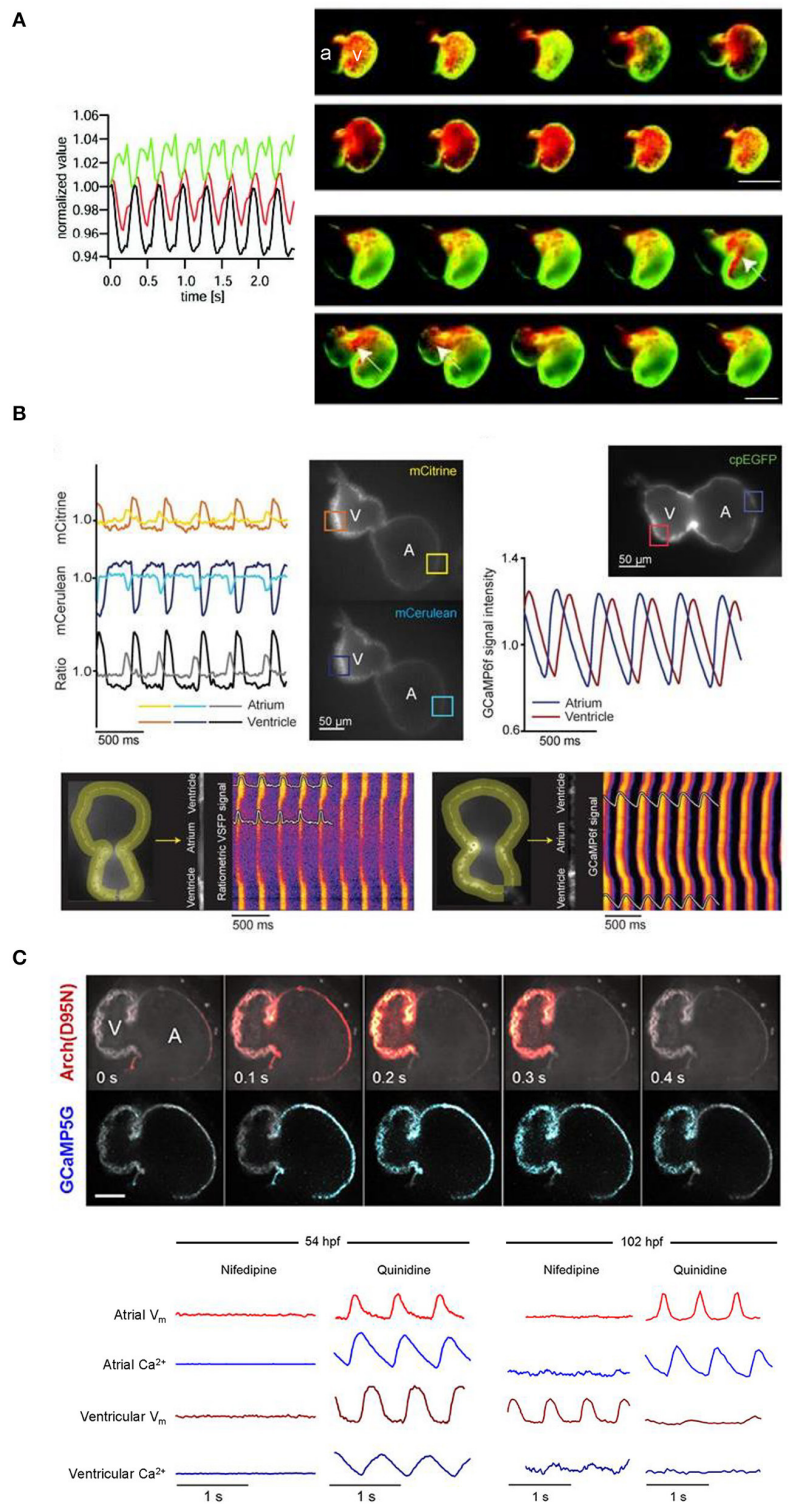
*In vivo* imaging of membrane potential using genetically encoded voltage indicators (GEVIs), combined with genetically encoded calcium indicators (GECIs) in intact zebrafish embryos. **(A)** Signals (left) from a donor (mUKG, green) and acceptor (mKOκ, red) Förster resonance energy transfer (FRET) pair of fluorescent proteins and their ratio (mKOκ/mUKG, black) acquired from the ventricle of a 3 days post-fertilization (dpf) zebrafish embryo with cardiac-specific expression of the GEVI Mermaid. Pseudo-colored ratio images (right) representing a single cardiac cycle in wild-type, Mermaid-expressing zebrafish (upper) showing propagation of excitation from the sinus venosus in the atrium (a) to the ventricle (v), and in astemizole-treated (5 μM, 15 min) zebrafish (lower) showing retrograde propagation from the ventricle to the atrium (highlighted with arrows). Scale bar, 100 μm. From Tsutsui et al. (2010). **(B)** Signals (upper left) from a donor (mCerulean, blue) and acceptor (mCitrine, yellow) Förster resonance energy transfer (FRET) pair of fluorescent proteins and their ratio (mCitrine/mCerulean, black) acquired from the regions of interest (boxes in fluorescent images) indicated on the atrium (A) and ventricle (V) of a 3 dpf zebrafish embryo with cardiac-specific expression of GEVI VSFP-butterfly CY. Signals (upper right) acquired from the atrium (blue) and ventricle (red) of a 3 dpf zebrafish embryo with cardiac-specific expression of the GECI GCaMP6f. Line plots of chimeric VSFP-butterfly CY (lower left) and GCaMP6f (lower right) background-corrected fluorescence intensities averaged across the width of the myocardial wall superimposed on heat maps of trajectory vs. time illustrating electrical impulse and Ca^2+^ propagation throughout the heart. cpEGFP, circularly permutated enhanced green fluorescent protein. From van Opbergen et al. (2018a). **(C)** Single optical sections of a 4 dpf zebrafish heart with cardiac-specific expression of the GEVI-GECI construct CaViar showing GEVI Arch(D95N) (top) and GECI GCaMP5G (middle) fluorescence as excitation propagates from the atrium (A) to ventricle (V). Voltage (V_m_, red) and calcium (Ca^2+^, blue) signals acquired from the atrium and ventricle of 50 (upper left) and 102 (lower right) hours post-fertilization (hpf) zebrafish embryos exposed to nifedipine (L-type Ca^2+^ channel blocker) and quinidine (fast sodium channel blocker). At 52 hpf, nifedipine reversibly suppressed voltage and Ca^2+^ dynamics in both chambers, while quinidine had no effect. At 102 hpf, nifedipine largely suppressed calcium transients in both chambers but only suppressed atrial voltage, while quinidine largely suppressed voltage and calcium transients in the ventricle but did not affect either transient in the atrium. Scale bar, 50 μm. From Hou et al. (2014).

The development of effective GEVIs has been slower than GECIs, owing to difficulties in achieving sufficiently fast kinetics and avoiding electrophysiological interference. Recently, significant progress has been made, which includes their application for cardiac research. The first reported use of GEVIs in the heart was in fact in zebrafish (Tsutsui et al., 2010), which utilized a FRET-based voltage-sensitive fluorescent protein (VSFP) called Mermaid (Tsutsui et al., 2008). The Mermaid construct consists of a green-emitting fluorescent donor (mUKG; *Umi-Kinoko* from *Sarcophyton*) and an orange-emitting fluorescent acceptor (mKOκ; *Kusabira* from *Fungia concinna)* fused to a voltage sensing phosphatase from *Ciona intestinalis* (Ci-VSP) with a transmembrane domain homologous to the S1–S4 segments of voltage-gated potassium (Kv) channels (Murata et al., 2005). The Mermaid reporter was expressed specifically in the zebrafish heart under the *cmcl2* promotor [*Tg(cmlc2:Mermaid)*] and used for *in vivo* voltage mapping in 2–3 dpf embryos under normal conditions and after application of the histamine H1 receptor blocker astemizole (known to also block *I*_Kr_). Measurements showed that astemizole disrupted the normal sequence of cardiac excitation, causing retrograde propagation from the atrioventricular boundary to the atrium (Figure 4A) (Tsutsui et al., 2010).

Since that time, a variety of new GEVIs have been developed with improved sensitivity and kinetics. In a recent study using zebrafish (van Opbergen et al., 2018a), the novel GEVI chimeric VSFP-butterfly CY (cyan-yellow, mCitrine/mCerulean) (Mishina et al., 2014) or the updated GECI GCaMP6f (Chen et al., 2013) were expressed in the heart of pigment-deficient, optically-transparent *casper* mutant zebrafish (White et al., 2008), with the myosin light chain 7 (*myl7*) promotor [*Tg(myl7:chimeric VSFP-butterfly CY)* and *Tg(myl7:Gal4FF; UAS:GCaMP6f)*]. The hearts of 3 dpf and 14 dpf zebrafish were imaged after administration of drugs targeting the sympathetic nervous system or various cardiac ion channels to assess effects on electrical activation, action potential morphology, and intracellular Ca^2+^ dynamics (van Opbergen et al., 2018a) (Figure 4B). It was shown that: (i) β adrenergic receptor stimulation (with isoproterenol) or blockade (with propranolol) increased or decreased diastolic Ca^2+^ levels and Ca^2+^ transient amplitudes, respectively; (ii) *I*_Kr_ block (with E-4031) increased action potential duration; (iii) L-type calcium current (*I*_Ca, L_) block (with nifedipine) prevented Ca^2+^ transients, increased ventricular action potential duration, and disrupted atrioventricular conduction; and (iv) differences exist in atrial and ventricular Ca^2+^ recovery dynamics between 3 and 14 dpf zebrafish (but not in the Ca^2+^ upstroke).

### Combined Voltage-Ca^2+^ Imaging

Functional fluorescent dyes can be combined for simultaneous mapping of voltage and Ca^2+^ in the whole heart (Herron et al., 2012). There is similar interest in combining GEVIs and GECIs for dual voltage-Ca^2+^ imaging, however this is generally prevented by spectral overlap of the relevant fluorescent proteins. The first successful study using a GEVI-GECI construct in the heart was performed in the zebrafish, using a genetically encoded dual-function voltage-Ca^2+^ reporter (“CaViar,” created by fusing the GEVI Arch(D95N) with the GECI GCaMP5) under control of the heart-specific *cmlc2* promoter [*Tg(cmlc2:Arch(D95N)-GCaMP5G)*] (Hou et al., 2014). Hearts of 1.5–4.5 dpf embryos were imaged during application of the *I*_Ca, L_ blocker nifedipine or fast sodium channel blocker quinidine, which showed that early in development the zebrafish cardiac AP is initiated by Ca^2+^, but by 4 dpf the ventricular AP becomes driven by sodium, while the atrial AP remains Ca^2+^ dependent (Figure 4C).

## Studies Utilizing Optogenetic Actuators in the Zebrafish Heart

**Figure 5:**
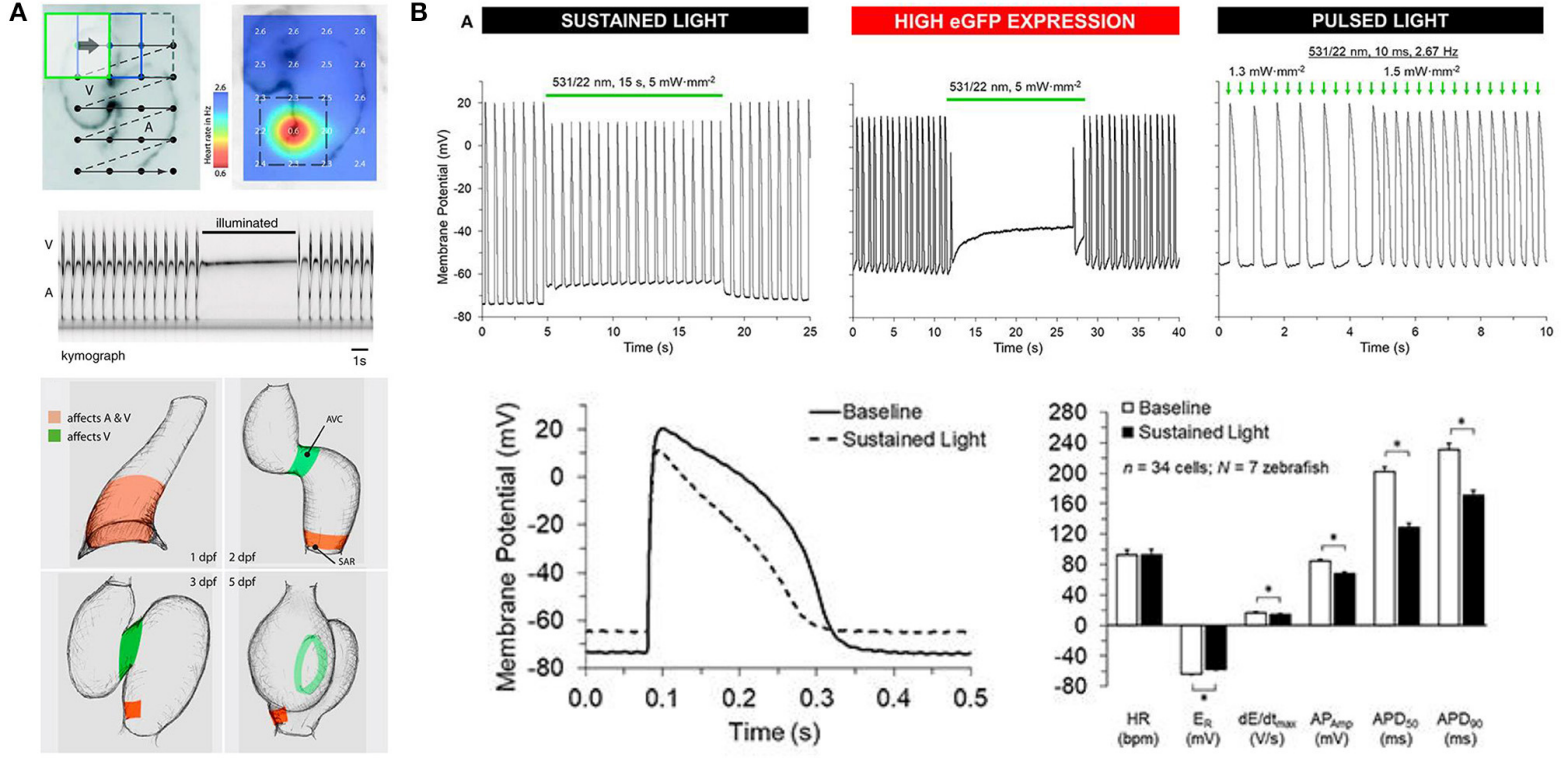
Optogenetic manipulation of membrane potential in zebrafish hearts. **(A)** *in vivo* stimulation of halorhodopsin by patterned illumination of 3 days post-fertilization (dpf) embryonic hearts (upper left) reveals the location of the pacemaker by a reduction in heart rate (**upper right**). Illumination of the whole heart could stop atrial (A) and ventricular (V) beating (middle). Areas that control atrial and ventricular (red) or only ventricular (green) contractions were revealed throughout embryogenesis (1–5 dpf, bottom). From Arrenberg et al. (2010). **(B)** Sustained stimulation of *Guillardia theta* anion channelrhodopsins 1 (GtACR1) by spot illumination on the ventricle of 3 months post-fertilization zebrafish isolated hearts caused an immediate increase in resting membrane potential (ER) and a decrease in the maximum rate of membrane depolarization (dE/dt_max_), AP amplitude (AP_Amp_), and APD at 50% and 90% repolarization (APD_50_ and APD_90_, upper left and bottom). In the case of particularly high GtACR1 expression (represented by high eGFP expression), ventricular excitation could be silenced (upper middle). Pulsed light, on the other hand, could be used to stimulate the heart (upper right). *Indicates *p* < 0.0001 by two-tailed paired Student's *t*-test. From Kopton et al. (2018).

Optogenetic actuators are light-activated proteins that generate a transmembrane ion flux. The discovery and cloning of the cation-selective ion channel channelrhodopsin-2 (ChR2) from the green alga *Chlamydomonas reinhardtii* in 2003 (Nagel et al., 2003) has led to the development of an extensive toolkit that includes depolarizing (excitatory) and hyperpolarizing (inhibitory) opsins, which are activated across a wide spectrum of wavelengths, and may be used for manipulation of cardiac membrane potential (Figure 1) (Schneider-Warme, 2018; Ferenczi et al., 2019; Entcheva and Kay, 2021). As for the cardiac application of GEVIs and GECIs, one of the first reports of the use of optogenetic actuators in the heart was in zebrafish (Arrenberg et al., 2010). This involved the use of both ChR2 [*Tg(E1b:Gal4*^*s*1101*t*^*; UAS:ChR2(H134R)-eYFP*^*s*1990*t*^*)*] and the chloride-specific ion channel halorhodopsin from *Natronomonas pharaonis* (NpHR) (Zhang et al., 2007) [*Tg(E1b:Gal4-VP16*^*s*1101*t*^*; UAS:NpHR-mCherry*^*s*1989*t*^*)*], to locate and control cardiac pacemaker cells in intact 1–5 dpf zebrafish embryos. In NpHR-expressing zebrafish, maps were generated at each day post-fertilization by sequentially illuminating small, overlapping regions of the heart, and measuring the heart rate response, or the incidence of cardiac arrest or arrhythmia (Figure 5A). It was found that: (i) at 1 dpf, the heart stopped beating when a region at the venous pole was illuminated, indicating the location of the pacemaker; (ii) at 2 dpf, the pacemaker region was more confined to the sinoatrial ring, with illumination of large adjacent areas having no effect, and atrioventricular block (of varying degree, depending on light intensity) occurred with illumination of the atrioventricular canal; and (iii) at 3 dpf, the pacemaker region was more defined, being confined to the dorsal right quadrant of the sinoatrial ring. It was further found that in 4 dpf embryos, pulsed photo-stimulation of the sinoatrial ring at a frequency of 2.7–4.7 Hz with ChR2 was able to control heart rate.

Since the time of that pioneering study, another prominent family of chloride-specific light-activated ion channels has been developed, the *Guillardia theta* anion channelrhodopsin 1 and 2 (GtACR1 and GtACR2) (Govorunova et al., 2015), which have been shown to silence neuronal AP generation (including in zebrafish) (Malyshev et al., 2017; Mauss et al., 2017; Mohamed et al., 2017; Forli et al., 2018). The first cardiac application of GtACR1 involved zebrafish [using hearts isolated from 3 months post-fertilization adults with cardiac-specific GtACR1 expression, *Tg(cmlc2:GtACR1-eGFP)*], which in combination with experiments in genetically transfected single rabbit ventricular myocytes demonstrated that GtACR1 activation causes depolarization of ventricular myocytes when applied during the resting (diastolic) phase of the AP (and if suprathreshold, results in excitation), but causes repolarization when applied during the (systolic) plateau (resulting in shortening of the AP). This biphasic response relates to the reversal potential of chloride in ventricular myocytes, which is somewhere between −40 and −33 mV (Clemo et al., 1999), so that the flow of negative ions switches from outward (causing depolarization) to inward (causing repolarization) as cells are excited. As a result, pulsed illumination can be used to pace the heart, while sustained illumination can arrest the heart in a depolarized state (Figure 5B) (Kopton et al., 2018). This indicates that while GtACR1 does not address the need for optogenetic silencing through a physiological means (i.e., hyperpolarization), it is a potentially attractive tool for exciting cardiomyocytes by transient light-induced depolarization.

## Future Directions for the Use of Cardiac Optogenetics in Zebrafish

Unlike the prevalent use of optogenetics in zebrafish to study the nervous system (Del Bene and Wyart, 2012; Simmich et al., 2012; Portugues et al., 2013), there have been relatively few cardiac optogenetic studies performed in zebrafish (summarized in Table 2), yet those studies have been fundamental in progressing the application of optogenetic technologies to the heart. With the continual improvement of optogenetic techniques (Entcheva and Kay, 2021), the use of zebrafish for integrative (patho-)physiological cardiac structure-function studies holds great promise. Future research will be driven by technological advances such as high-speed, cell-accurate, three-dimensional mapping (Mickoleit et al., 2014; Weber et al., 2017; Sacconi et al., in press), more effective methods for cell-specific spatial and temporal gene expression (Reade et al., 2017; LaBelle et al., 2021), and novel optogenetic actuators and reporters with enhanced expression, fluorescence, and kinetics, combined with improved light delivery (Entcheva and Kay, 2021). As the field continues to develop, the zebrafish may be invaluable for cardiac optogenetic studies directly related to its strengths as an experimental model (Table 1; i.e., development, genetic screening, drug discovery, cardiotoxicity testing, disease modeling, all-optical studies of electrophysiology and cell signaling, and anti-arrhythmic strategy development). It may also be a powerful tool for fundamental investigations of the hetero-cellular heart (e.g., structure-function interactions of myocytes, fibroblasts, intracardiac neurons, and immune and endothelial cells) and in helping to overcome hindrances related to the clinical translation of optogenetic techniques (e.g., genetic transfection, immune responses, phototoxicity) (Richter and Bruegmann, 2020). This will be enhanced by the large number of currently available transgenic lines, genetic material, and tools (easily found through online resources and central repositories), facilitated by the open zebrafish community willing to share them (Rafferty and Quinn, 2018; Stoyek et al., in press).

While a majority of cardiac optogenetic studies in the zebrafish have been performed in the early stages of development, technological advances in fluorescence imaging approaches and methods for spatially-resolved light stimulation have the promise to enable studies to be performed in the adult isolated whole heart and *in vivo*. This will be aided by the continuing development of transgenic lines that lack pigment and are thus largely transparent throughout their lifespan (i.e., “casper” [White et al., 2008) and “crystal” (Antinucci and Hindges, 2016)], and can be used as a background on which to express optogenetic reporters and actuators along with mutations of interest.

## Conclusion

Optogenetics is a powerful and highly successful (Deisseroth, 2010) set of techniques that has been instrumental in recent developments in neuroscience research (Deisseroth, 2015; Kim et al., 2017), and more recently also for cardiac research (Entcheva and Kay, 2021). Zebrafish provide specific advantages as an experimental model for optogenetic cardiac investigations (Gut et al., 2017; Stoyek and Quinn, 2018), and have been instrumental in its early development, suggesting a bright future for this little fish. With the increasing sophistication of optogenetic methods, the zebrafish represents an experimental model with great potential for cardiac optogenetic studies. Hopefully more cardiac researchers will soon begin to see the light.

## Author Contributions

JB wrote the manuscript. MS and TQ revised the manuscript. All authors approved the final version.

## Funding

This work was supported by the Natural Sciences and Engineering Research Council of Canada (RGPIN-2016-04879 to TQ), the Heart and Stroke Foundation of Canada (G-18-0022185 to TQ), and the Canadian Institutes of Health Research (MOP 342562 to TQ).

## Conflict of Interest

The authors declare that the research was conducted in the absence of any commercial or financial relationships that could be construed as a potential conflict of interest.

## Publisher's Note

All claims expressed in this article are solely those of the authors and do not necessarily represent those of their affiliated organizations, or those of the publisher, the editors and the reviewers. Any product that may be evaluated in this article, or claim that may be made by its manufacturer, is not guaranteed or endorsed by the publisher.
